# Predictors of fulvestrant long-term benefit in hormone receptor-positive/HER2 negative advanced breast cancer

**DOI:** 10.1038/s41598-022-16409-7

**Published:** 2022-07-27

**Authors:** Rosalba Torrisi, Valentina Vaira, Laura Giordano, Annarita Destro, Vera Basilico, Saveria Mazzara, Piermario Salvini, Gabriella Gaudioso, Bethania Fernandes, Noemi Rudini, Giovanna Masci, Armando Santoro

**Affiliations:** 1 Department of Medical Oncology and Hematology Unit, IRCCS Humanitas Research Hospital, via A. Manzoni 56, 20089 Rozzano, MI Italy; 2 Division of Pathology, Fondazione IRCCS Ca’ Granda-Ospedale Maggiore Policlinico, Milan, Italy; 3 Department of Pathophysiology and Transplantation, University of Milan, Milan, Italy; 4 Pathology Department, IRCCS Humanitas Research Hospital, Rozzano, MI Italy; 5 Medical Oncology Unit, Istituto Clinico Mater Domini Humanitas, Castellanza, VA Italy; 6 Division of Haematopathology, IEO European Institute of Oncology IRCCS, Milan, Italy; 7 Medical Oncology, Humanitas Cliniche Gavazzeni, Bergamo, Italy; 8 Department of Biomedical Sciences, Humanitas University Pieve Emanuele, Milan, Italy; 9 IRCCS Humanitas Research Hospital- Humanitas Cancer Center, Rozzano, MI Italy

**Keywords:** Cancer, Molecular biology

## Abstract

We retrospectively investigated in women treated with fulvestrant for HR+/HER2 negative advanced breast cancer clinical, pathological and molecular features associated with long-term benefit from treatment defined as being progression-free at 18 months. Specifically, we analyzed on formalin-fixed paraffin-embedded tumor samples *ESR1* and *PI3KCA* mutations and miRNAs profiles. 59 patients were evaluable (median age of 67 years, range 32–92). 18-month PFS rate was 27%; the lack of visceral metastases significantly predicted the likelihood of being progression-free at 18 months, while *PI3KCA* mutations, found in 36% of patients, were not associated with 18-month PFS. As of miRNAs, *miR-549a*, *miR-644a, miR-16-5p* were negatively while *let-7c-5p* was positively associated with 18-month PFS. In addition*, miR-520d-3p* and *miR-548g-3p* values were significantly lower while *miR-603, miR-181a-5p* and *miR-199a-miR-199b-3p* values were significantly higher in patients achieving 18-month PFS. In silico analysis of targets modulated by these two latter groups of miRNAs show that in patients achieving 18-month PFS the Hippo and Wnt signaling pathways were predicted to be upregulated while endocrine resistance was potentially repressed by *miR-603, miR-181a-5p* and *miR-199a-miR-199b-3p.* Our results provide additional clues on the molecular mechanisms involved in fulvestrant activity and resistance. Underlying pathways should be further elucidated and confirmed in larger cohorts.

## Introduction

About two thirds of breast cancers are hormone receptor-positive HER2 negative (HR+/HER2−)^1^. The combination of endocrine therapy (ET) and CDK4/6 inhibitors (CDK4/6i) has established as standard treatment for HR+/HER2− advanced breast cancer (ABC)^2^. Four randomized trials including thousands of patients have shown an impressive and consistent advantage of 10–14 months in progression free survival (PFS) for the association of any of the three CDK 4/6i (palbociclib, ribociclib and abemaciclib) investigated with non-steroidal aromatase inhibitors (NSAI) in endocrine sensitive ABC as compared to the endocrine agent alone^3^.

On the other hand, since multiple mechanisms of resistance underlying CDK4/6i failure have been proposed, no definite treatment indications after progression to CDK4/6i exist and international guidelines indicate that a further endocrine manipulation as well as a switch to chemotherapy may be offered^2,4^.

The Selective Estrogen Receptor Downregulator (SERD) fulvestrant has established as a valuable option in the treatment of advanced HR+/HER2− ABC. Head-to-head comparison with NSAI in the 1st line treatment has shown the superiority of fulvestrant^5^ and in the CONFIRM study NSAI-resistant patients achieving a clinical benefit with fulvestrant had a prolonged disease control (> 16 months)^6^.

Thus it appears crucial to identify clinical and pathological factors which may predict the subset of patients who maintain endocrine sensitiveness and may be spared more aggressive treatments for a clinically relevant span of time and, on the other hand, patients who are likely to derive no or minimal benefit from fulvestrant and who deserve more effective treatments upfront. At the present time no predictors of response or resistance have been established.

MicroRNAs (miRNAs) are a class of small, non-coding RNA that regulate the gene expression of target mRNAs at post-transcriptional level^7,8^. miRNAs have been implicated in regulating breast cancer cell proliferation, cell death, apoptosis, immune response, cell cycle energetics, senescence, invasion, and metastasis^9^. Moreover, miRNAs are emerging as novel potential predictive/prognostic biomarkers of disease and response to therapies^8,9^. Several miRNAs have been found deregulated in breast cancer cell lines (BCCL), on cancer specimens and in serum of breast cancer patients and have been correlated with prognostic features and with response and resistance to endocrine manipulations^9^.

In the present study we investigated in a series of women with HR+/HER2− ABC treated with fulvestrant whether a panel of miRNAs was associated with long-term benefit from fulvestrant. According to previous literature data long-term benefit was defined as treatment duration without progression lasting at least 18 months^10^.

In addition, since endocrine resistance has been associated with mutations of *ESR1* and *PI3KCA* the occurrence of these mutations was assessed and correlated with miRNA profiles and with outcome^11,12^.

## Patients and methods

This observational retrospective multicentric study which aimed to describe the clinical outcome of patients with HR+/HER2− ABC who received fulvestrant as part of their routine treatment at 3 Italian Institutions and to correlate it with clinical, pathological and molecular features.

Eligible patients were pre/peri- and postmenopausal women aged ≥ 18 years diagnosed with HR+ (defined as ER and/or PgR ≥ 10%) and HER2− (defined as IHC 0, 1 + or 2 + with FISH negative) inoperable locally advanced and/or metastatic breast cancer for whom a tissue specimen of advanced or metastatic disease obtained for diagnosis was available. Pre/perimenopausal patients received concomitant ovarian suppression with GnRH analogues. Fulvestrant was administered 500 mg i.m. q28 days with loading dose after 14 days as any line of treatment. Patients treated before October 2011 received fulvestrant 250 mg i.m q28 days.

All tumor samples were obtained after diagnosis of advanced and/or metastatic disease and before fulvestrant treatment and were analyzed for routine diagnosis at the same pathologic department by breast dedicated pathologists.

Patient medical charts were reviewed and tumor response was assessed. Since this is a retrospective study response could not be evaluated according to RECIST 1.1 criteria in all patients, but CT scans and other imaging were reviewed in order to comply with RECIST criteria whenever possible. Information on age at diagnosis, menopausal status, previous adjuvant therapies, previous therapies for metastatic disease, pathological features of the metastatic tumor, sites of metastatic disease, best response, and disease progression if occurred and last follow up visit were collected.

All patients signed informed consent for participation to the study.

This study was approved by the institutional ethical committee of the coordinating center (Humanitas Research Hospital) and of the other participating Institutions and was conducted in compliance with 1964 Helsinki Declaration.

### Mutation analyses

DNA was extracted from 5 slides (5 μm thickness) paraffin-embedded breast cancer tissue at least 70% tumor enriched using the Maxwell RSC DNA FFPE Kit (Promega) according to the manufacturer’s instructions. Manual microdissection was performed to precisely separate tumor regions from normal tissue. The enrichment in tumor cells reached a level higher than 80% upon microdissection. DNA was quantified by Nanodrop 2000 spectrophotometer (NanoDrop Products, Wilmington, DE, USA). Both *PIK3CA* (E542K, E542Q, C420R, H1047P, H1047R, H1047L, E545K, E545Q, Q546K, Q546E, E545A, E545G, E545V) and *ESR1* (E380Q, S463P, D538G, D538V, L536H, L536R, L536P, Y537C, Y537S, Y537N, P535H) hot spot mutations were analyzed by processing 50 ng DNA using the Sequenom MassARRAY platform (SEQUENOM^®^ Inc). The sequences and molecular weights of extension primers and products were listed in Supplementary Table 1. A cutoff of 5% of allelic frequency was generally guaranteed.

### miRNA analyses

Tumor enriched RNA was extracted through manual microdissection of 5 paraffin-embedded breast cancer tissue slides (5um thickness) per patient as previously described for DNA. Total RNA isolation using the using the Maxwell RSC RNA FFPE kit (Promega, Madison, WI, USA) according to the manufacturer’s protocol. Quantification and RNA quality assessment were performed using a Nanodrop 2000 spectrophotometer (NanoDrop Products, Wilmington, DE, USA).

miRNA expression profiling was performed using the nCounter Human v3 miRNA Panel (NanoString Technologies, Seattle, WA, USA), which contains 798 unique miRNA barcodes. RNA was prepared according to the manufacturer’s protocol in a total of 5 runs. The nCounter Flex instrument was used and all counts were gathered by scanning on HIGH mode for 555 fields of view per sample. Raw data were analyzed as described in nCounter Data Analysis Guidelines for miRNA^13^. Specifically, the background threshold was set at 20; any value below this threshold was converted to zero. After that, miRNA counts were normalized to the geometric mean of the 50 miRNAs with the highest counts. Then, normalized miRNA values were subjected to statistical analyses. Nanostring data processing was carried out using R 3.6.1 software (R Core Team)^14^.

Predicted miRNA targets were searched using the miRTargetLink2 web tool, selecting only validated targets. Then, miRNAs targets were imported in STRING database and analyzed for significantly enriched Gene Ontology (GO) terms (Biological process and Molecular function) and Kyoto Encyclopaedia of Genes and Genomes (KEGG) Pathways using the human whole genome as statistical background as previously described (10.3389/fonc.2021.643280).

### Statistical analyses

Categorical variables were expressed as number and percentages, while continuous variables as medians with the respective range.

Clinical and demographical data and miRNAs were associated with outcome as continuous value in terms of progression free survival (PFS) by the cox proportional hazard model to calculate hazard ratio and their corresponding 95% confidence intervals. Moreover, the impact of miRNAs with long-term survival was described considering as success patients with a PFS ≥ 18 months and estimating Odds ratios with their corresponding 95% confidence intervals with a logistic regression model. Distribution of miRNAs between patients achieving or not 18-month PFS were estimated by Wilcoxon t test. For patients presenting miRNA assessment both at baseline (miRNAto) and at progression (miRNApd), a normalized variation was calculated as (miRNAto − miRNApd)/miRNAto to explore a possible miRNA profile association with progression.

Considering the small sample size all evaluation were explorative in nature. In the analysis of the 118 miRNAs the adjusted α level should be equal to 0.00042 considering Bonferroni Correction. A statistical p value of 0.05 was arbitrarily set to identify miRNAs of potential interest to be further explored in a larger sample size.

All analyses were performed using SAS version 9.4 (SAS Institute Inc, Cary, NC).

## Results

From a database of 202 women with HR+/HER2− ABC who received treatment with fulvestrant from 2005 to 2017 at the 3 participating Institutions were retrieved 74 patients who underwent a diagnostic biopsy for advanced or metastatic disease before treatment. Fourteen patients were excluded because there was no residual tissue for further analyses and one additional patient did not have clinical information. Fifty-nine patients were evaluable for the molecular analyses. Tumor tissue was obtained from pleura (16), breast, when biopsy of synchronous metastatic sites was not feasible (15), lymph nodes (9), bone (7), skin (5), liver (4), other sites (3). Seven patients had also a biopsy obtained after progression on fulvestrant.

Baseline patient characteristics are summarized in Table 1. Median age was 67 years (range 32–92).

**Table 1:** Patient and tumor characteristics.

	N	%
Age in years, median (range)	67	(32; 92)
**Menopausal status, n (%)**		
Pre/peri-menopausal	15	25.4
Post-menopausal	43	72.9
Unknown	1	1.7
**HR status, n (%)**		
ER+/PgR+	34	57.6
ER+/PgR−	24	40.7
Unknown	1	1.7
**HER2 status, n (%)**		
Negative	51	86.4
Positive	5	8.5
Unknown	3	5.1
**Ki67**		
≤ 20	24	40.7
> 20	24	40.7
Unknown	1	1.7
**Tumor subtype**		
Luminal A	24	40.7
Luminal B	27	45.8
Luminal HER2	3	5.1
Unknown	5	8.5
**Metastatic sites (number)**		
≤ 3	54	91.5
> 3	5	8.5
**Metastatic sites**		
Bone-only	9	15.3
Visceral	28	47.5
Other	22	37.3
**Previous therapy with NSAI**		
Metastatic setting	12	20.3
Adjuvant setting	19	32.2
Both	1	1.7
Missing	27	45.8
**Prior lines of therapy for metastatic disease**		
0	8	13.6
1	10	16.9
2	13	22.0
≥ 3	27	45.8
Unknown	1	1.7
**Status at fulvestrant**		
Progressive disease	48	81.4
Response or stable disease	11	18.6

### Clinical results

Overall median PFS (mPFS) was 10.7 months (range 2.2–14) and clinical benefit was obtained in 36 patients (61%). No clinical variable (tumor subtype, number of metastatic sites, number of previous lines, disease status at fulvestrant) was statistically significantly associated with mPFS, although a trend towards a shorter mPFS in patients with visceral metastases vs patients with no visceral involvement was observed (7.5 months vs 12.6, respectively p = 0.059). At the same time, patients with both HR positive tumors had a not statistically significant longer PFS as compared with patients with PgR negative tumors (13.8 vs 7.5 months, respectively, p = 0.099).

At 18 months 16 patients were progression free and alive with an 18-month PFS rate of 27%. Analyses of clinical and pathological variables showed that only the lack of visceral metastases significantly predicted the likelihood of being progression-free at 18 months (OR 0.25, 95% CI 0.07–0.9 p = 0.035).

Nonetheless, patients with ≤ 3 metastatic sites (OR: 1.6, CI 95% 0.2;14.9; p value: 0.71), patients with Luminal A tumors (OR 1.4, CI 95% 0.4; 4.8; p = 0.563), and patients treated with < 3 previous lines of therapy (OR 1.7, CI 95% 0.4–5; p = 0.390) had a higher probability to be alive and progression-free at 18 months as compared with patients with > 3 metastatic sites, with luminal B tumors and treated with ≥ 3 lines, respectively, although these results did not reach statistical significance.

### Mutation analyses

*ESR1* and *PI3KCA* mutations were assessed in 53 patients. *ESR1* mutations were observed in the pretreatment biopsy in 5 patients, all previously treated with NSAI: D538V and Y537S (2 patients) and E380Q (1 patient). In the 7 patients with repeated biopsies *ESR1* mutations were observed in both biopsies in 1 patient (Y537S) while they were detected only in the post-progression biopsy in 2 cases (Y537S and D538G). Since *ESR1* mutations were found only in 9% of the patients, no correlation with other features was investigated.

*PI3KCA* mutations were found in pretreatment biopsies in 19 (36%) patients: H1047R (10 patients), E542K (4 patients), E545K and H1047L (2 patients), Q546K (1 patient). Among the 7 patients with repeated biopsies only 2 patients had *PI3KCA* mutations in both samples while 1 patient lost the *PI3KCA* mutation and developed an *ESR1* mutation in the post-progression biopsy.

PFS in patients with *PI3KCA* mutated tumors was numerically shorter than in patients not harboring mutations (mPFS 7.6 vs 11 months, respectively, p = 0.28) On the other hand, the probability of being progression-free at 18 months was not different in the 2 groups (OR 1.01, 95% CI 0.28–3.6 p = 0.99).

### miRNA analyses

miRNA profiles were assessed in 50 patients since patients with bone biopsies were excluded for this analysis because of poor RNA quality.

45 miRNAs were detected in at least 30 samples, 83 miRNAs were detected in at least 20 samples and 118 miRNAs were detected in at least 10 samples.

We investigated association with outcome only for miRNAs which were detected in at least 20% (n = 10) of samples (detailed in Supplementary Table 2).

miRNAs which were statistically significant associated with PFS are reported in Table 2. Fourteen miRNAs were negatively associated with outcome while 2 miRNAs belonging to the let-7 family were positively associated with improved outcome.

**Table 2:**
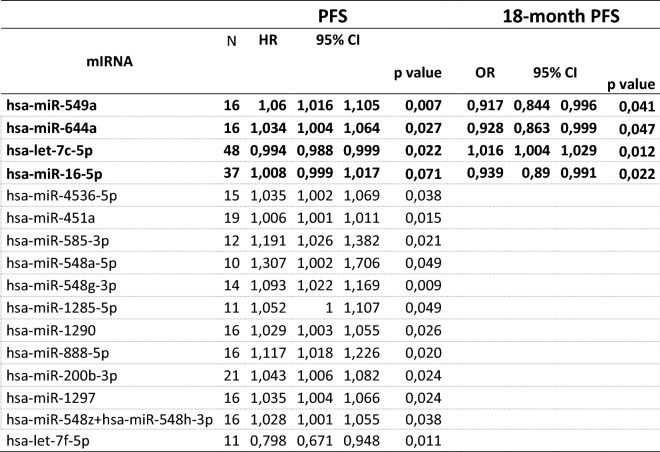
miRNAs associated with outcome.

*PFS* progression-free survival, *HR* hazard ratio, *95% CI* 95% confidence interval, *OR* odd ratio.

In bold miRNA which are statistically significantly associated with 18-month PFS (p < 0.05).

When we considered the likelihood of being progression free at 18 months as endpoint only *miR-549a*, *miR-644a* confirmed to be negatively associated with outcome in addition to *miR-16-5p*, while *let-7c-5p* was associated also with a greater likelihood of being progression free at 18 months (Table 2).

When we analyzed miRNA distribution comparing patients with or without a PFS ≥ 18 months we found that *miR-549a*, *miR-644a, miR-16-5p* values were significantly higher while *let-7c-5p* value was lower in patients not achieving 18-month PFS, respectively. In addition to these*, miR-520d-3p and miR-548g-3p* were significantly and negatively associated with 18-month PFS while *miR-603, mir-181a-5p* and *miR-199a-miR-199b-3p* values were statistically significantly higher in the long-term benefit cohort; moreover *miR-125b-5p* showed a not significant trend to be higher in patients being progression free at 18 months. No statistically significant results were found based on adjusted p value (Bonferroni correctioni, p = 0.00042).

In silico analysis of predicted targets (Supplementary Tables 3, 4) showed that cell metabolism was the main biological process involved in fulvestrant treatment. Analysis of predicted pathways suggested that *miR-520d-3p* and *miR-548g-3p* decrease might induce Hippo and Wnt signaling (Supplementary Table 5). On the contrary, high levels of *miR-603, mir-181a-5p, miR-199a-miR-199b-3p* might repress endocrine resistance thus potentially promoting the anti-tumor activity of Fulvestrant (Supplementary Table 6).

Finally, no specific miRNA expression was associated with *PI3KCA* mutation status (data not shown). Similarly, we did not find any specific baseline miRNA profile or change at disease progression in the 7 patients for whom both a baseline and post progression biopsy were available (data not shown).

## Discussion

The identification of clinical, pathological and molecular features predicting the benefit of treatments is a major goal for medical oncologists.

Fulvestrant has been proven a valuable option for treatment of HR+ ABC. First-line fulvestrant was associated with a mPFS of 16.6 months which extended to 22 months in patients with bone-only metastases in a population of mostly treatment-naïve patients (5). In the cohort of patients treated as 1st line in the MonaLEEsa-3 study mPFS in the Fulvestrant/placebo arm was 19 months, while in the control arms of 2nd/3rd line studies with CDK 4/6i and Fulvestrant, mPFS decreased to 9 and 4.6 months, respectively^15–17^. Similar data have been reported in patients treated with Fulvestrant in real-world series^18–20^.

Identification of pathological and molecular features predicting benefit from treatment in tumor samples of patients treated with fulvestrant has been attempted previously^21,22^. The *Trans*CONFIRM, a translational analysis within the CONFIRM trial aimed to identify clinical-pathological features and molecular signatures in the primary tumors predicting response to fulvestrant, showed in the 112 samples analyzed that only PgR and HER2 expression and a signature of 37 genes were independently associated with PFS^21^. Of note, no correlation with *ESR1* mutations on primary tumors was found^21^. Christensen et al. investigated in mRNA extracted from 226 tumor samples of patients treated with fulvestrant the predictive value of a mathematical algorithm based on the expression of multiple genes (DRP)^22^. The DRP was associated although not significantly with outcome in patients treated in earlier lines and unexposed to previous adjuvant endocrine therapies^22^.

Our clinical data are comparable with those mentioned above, showing a mPFS of 10.6 months in a heavily pretreated cohort, since 46% of our patients were treated in 3rd or later lines. Only the occurrence of visceral metastases was associated with a worse outcome, although we cannot exclude that the correlations between HR and *PI3KCA* mutation status and outcome were not significant only because of the limited sample size. Similarly, only the absence of visceral metastases was significantly associated with a fourfold higher likelihood of long-term benefit from fulvestrant.

Notably, patients starting Fulvestrant after progressing to other therapies experienced a similar benefit as patients receiving treatment as maintenance after chemotherapy (mPFS = 9.9 months vs 10.8 months, respectively), similarly to what reported in a larger real-world series^20^.

The role of *PI3KCA* mutations in the mechanisms of resistance to fulvestrant is not clear. Results of the fulvestrant/placebo arms in studies with PI3K inhibitors conducted in patients harboring or not *PI3KCA* mutations are inconsistent^23^. While in the BELLE-2 study mPFS in patients with *PI3KCA* wild-type tumors doubled that of patients with *PI3KCA* mutant tumors, no difference was observed in the same comparisons within the BELLE-3 and SOLAR-1 studies^23^. In a recent analysis on ctDNA of patients included in the PALOMA 3 trial *PI3KCA* mutations, while being among the most common mutations at baseline, did not increase after progression and did not correlate with PFS^24^.

In our study *PI3KCA* mutations were found in about 36% of patients, consistently with what expected in Luminal breast cancers^25^ and were associated with a numerically shorter PFS, but not with a lower likelihood of being progression-free at 18 months. These findings are only partially consistent with the analysis of long-term benefit in the fulvestrant/placebo arm of the PALOMA 3 trial since in this study only 6% of patients harboring PI3KCA mutations vs 39% of patients with *PI3KCA* wild type tumors were treated for ≥ 18 months^10^.

Similarly to what observed in the PALOMA 3 study, the lack of PgR expression was associated with a lower likelihood of long-term benefit from Fulvestrant^10^.

The rate of *ESR1* mutations was lower than expected, despite more than 50% of patients had previously received a NSAI^25^. Notably *ESR1* mutations were observed only in post-progression biopsy in 2 patients, one of whom had lost at the same time *PI3KCA* mutation, suggesting that a different mechanism of resistance to fulvestrant was developed.

A huge number of miRNAs have been associated with breast cancer with alternative and not always consistent suppressive or oncogenic properties for each miRNA and neither meta-analyses have been able to define an unequivocal expression and role even in case of frequently expressed miRNAs^9,26–29^. Discrepancies among studies may be attributed to several factors as differences in patient populations, biological samples (fresh tissues, paraffin-embedded tissues and blood) and methodological procedures (qRT- PCR, NGS, microarray) but may also be related to different functions of each miRNA according to the tumor microenvironment^9,26–29^.

In our study we identified 9 miRNAs which were significantly and differently associated with 18-month PFS. Literature data provide evidence about 3 of these miRNAs (*let-7c*, *miR-520d-3p, miR-181a*) to be involved in HR+ ABC^30–35^. In particular *miR-520d-3p* was among miRNAs which were downregulated upon estradiol stimulation in BCCL^30^. On the other hand, *miR-520d-3p* has also been proposed to suppress *ESR1* expression and to be involved in endocrine resistance^31,32^.

A larger amount of consistent evidence is available on the *let-7* miRNA family which was demonstrated to target ER- alfa and negatively affect its function in ER-positive BCCL^33^. In particular, *let-7c* targets *ESR1* and is less expressed in metastatic tissue than in primary tumor and normal tissue^32^. Upon analysis of clinical data from The Cancer Genome Atlas (TCGA), it was suggested that low expression of *let-7c* in addition to other miRNAs (*miR-99a* and *miR-125b*) was associated with worse overall survival compared with patients who had high expression of these miRNAs^34^.

Our findings showing that *let-7c-5p* was significantly and positively correlated with a higher likelihood of being progression-free at 18 months and *let-7f-5p* significantly directly correlated with PFS confirmed the tumor suppressive properties for the members of the let-7 family.

In addition, we found a not significantly higher expression of *miR-125b* in patients achieving 18-month PFS, supporting the protective role outlined by TCGA for this miRNA^34^.

Contradictory evidence is available on *miR-181a-5p*, which belongs to a family of largely expressed miRNAs^29^. It was found to be upregulated and associated with poor survival in metastatic breast cancer patients particularly in those with TNBC^29^, but, on the other hand, it is among the most potent miRNAs repressing cell growth and counteracting estradiol-dependent cell proliferation^30^. In our study a greater expression of *miR-181a* was positively associated with long-term fulvestrant benefit.

Also on *miR-16-5p* literature data are somehow inconsistent since preclinical data support tumor suppressive properties but an increased expression in triple negative breast cancer (TNBC) as compared to normal tissues has been found as well as either a down- or upregulation in the serum of breast cancer patients as compared to healthy controls^33,35–37^. In our study *miR-16-5p* was associated with a decreased probability of being progression-free at 18 months.

In silico analysis of the targets of miRNAs inversely associated with long-term outcome showed that oncogene-induced cell senescence was the most affected biological processes. Analysis of predicted pathways suggested that *miR-520d-3p and miR-548g-3p* decrease might induce Hippo and Wnt signaling. On the other hand *miR-603* and *miR-181a-5p* and *miR-199a-miR-199b-3p* were predicted to suppress endocrine resistance.

The miRNAs we found significantly associated with long-term benefit or resistance to fulvestrant were not among miRNAs differently expressed in fulvestrant-resistant cell lines as compared with parental MCF-7 cells^38,39^. Moreover, in our series *miR-221* and *mir-222* which had been associated with fulvestrant resistance in BCCL were detected only in 2 and 8 samples, respectively and therefore were not further correlated with outcome; at the same time *miR-21,* despite being confirmed among the most highly expressed miRNAs, did not show any association with outcome (see Supplementary Table 2)^40,41^.

We acknowledge that our study has some limitations. First of all, the limited number of patients with available metastatic tissue which resulted in a small sample considering the large number of putative predictive variables examined especially for miRNA analyses; then the retrospective design which resulted in missing clinical information and which may have not allowed to meet with a strict application of RECIST 1.1 criteria for all patients. Moreover, we did not perform in vitro assays in BCCL to better elucidate the correlation between the miRNA profile associated with the in vivo response to fulvestrant and gene expression analyses to identify the related target genes involved. Finally, since the current use of fulvestrant monotherapy is recommended only after CDK 4/6i, we cannot rule out that this treatment can induce molecular patterns different from those observed in our CDK 4/6i naïve population.

In conclusion, altogether our clinical findings are consistent with literature data from randomized trials and real-world series, confirming the poor prognostic role of visceral metastases. Our data do not support an association between *PI3KCA* mutations and long-term benefit from treatment. Furthermore, we propose a new panel of miRNAs which were associated with long-term benefit of endocrine therapy and which are mainly involved in cell metabolism and endocrine resistance. Our results provide some additional clues on the mechanisms involved in fulvestrant activity and resistance although underlying molecular pathways should be further elucidated and confirmed in larger cohorts of breast cancer patients.

## Supplementary Information

Supplementary Tables.

## Data Availability

The datasets generated and analyzed during the current study are available from the corresponding author on request.
